# Treasurer's Report for Financial Year 2022

**DOI:** 10.1093/molbev/msad281

**Published:** 2024-01-04

**Authors:** John P McCutcheon, Jeffrey L Thorne

**Affiliations:** SMBE Treasurer; SMBE Treasurer

Society's Assets and Liabilities as of 2022 December 31:

**Table:** 

Assets	
Cash account	3,659,186.72$
Certificate of deposits	2,145,456.55$
Prepaid expenses	253,424.20$
Accounts receivable	0.00$
Total	6,058,067.47$
Liabilities	
Accounts payable	703,539.74$
Deferred membership	30,442.80$
Deferred Journal Revenue	576,732.00$
Total	1,310,714.54$
Net assets	4,747,352.93$

This report covers the 12 months from **2022 January 1** to **2022 December 31.** The Society continues to be in good financial health. For 2022, the net revenue collected by the Society was $821,734.14, with the main sources of revenue being our 2 journals, *Molecular Biology and Evolution* (*MBE*; $562,992) and *Genome Biology and Evolution* (*GBE*; $226,268). The revenue for both GBE and MBE was down from last year, but these 2022 figures will likely more accurately reflect the expected future revenue for the journals, as these figures reflect the complete shift to the Open Access model of publishing with no residual subscription carry-forward as has happened in the past 2 years.

Before the COVID-19 pandemic, most of the Society's expenses were dedicated to travel for the in-person annual meeting. However, the in-person annual meeting was again canceled in 2022 and was held virtually. The bulk of the Society's 2022 expenses of $723,018.30 were toward payouts to journal Editors-in-Chief, journal editing, and marketing efforts, the organization and implementation of the 2022 virtual meeting, and Society prizes such as the midcareer award, lifetime contribution award, and graduate student excellence awards. Some in-person SMBE Satellite meetings also returned in 2022.

Society's Revenues and Expenses for FY2022:

**Table:** 

Revenues	
Membership dues	22,076.56$
MBE FY2020 revenue	562,992.00$
GBE FY2020 revenue	226,268.00$
Unrestricted donation	495.00$
Interest income	9,902.58$
***Total revenues***	***821,734.14$***
Expenses	
Miscellaneous prize and career awards	11,000$
*Total prize and awards*	*11,000$*
Operating expenses	45,011.72$
Executive administration	36,595.95$
Corporation fee	40.00$
Council travel	112.38$
Banking charges	885.87$
Tax preparation and consultancy fees	5,500.00$
*Total operations*	*88,145.92$*
MBE editing	241,000.00$
Editorial assistance	7,796.62$
GBE editing	100,000.00$
GBE highlight honoraria	33,555.00$
*Total editorial*	*382,351.62$*
Annual SMBE meeting	72,843.28$
Seed money for 2023 meeting	22,471.48$
Satellite meetings	146,206.00$
*Total meetings*	*241,520.76$*
***Total expenses***	***723,018.30$***
***Change in net assets***	***98,715.84$***

